# The Food Source and Gut Bacteria Show Effects on the Invasion of Alien Pests—A Case of *Bactrocera dorsalis* (Hendel) (Diptera: Tephritidae)

**DOI:** 10.3390/insects15070530

**Published:** 2024-07-13

**Authors:** Yanfei Zhu, Rui Han, Tong Zhang, Jiawen Yang, Ziwen Teng, Yinjun Fan, Pengdong Sun, Yongyue Lu, Yonglin Ren, Fanghao Wan, Hongxu Zhou

**Affiliations:** 1 Shandong Engineering Research Center for Environment-Friendly Agricultural Pest Management, Shandong Province Centre for Bio-Invasions and Eco-Security, China-Australia Cooperative Research Center for Crop Health and Biological Invasions, College of Plant Health and Medicine, Qingdao Agricultural University, Qingdao 266109, China; yanfeizhu2021@163.com (Y.Z.); hanrui0506@126.com (R.H.); zero0852580@163.com (T.Z.); yangjiawen2024@163.com (J.Y.); tzwbat@126.com (Z.T.); fanyinjun89@126.com (Y.F.); wanfanghao@caas.cn (F.W.); 2 Special Food Research Institute, Qingdao Agricultural University, Qingdao 266109, China; pdsun02@163.com; 3 Department of Entomology, South China Agricultural University, Guangzhou 510642, China; luyongyue@scau.edu.cn; 4 College of Environmental and Life Sciences, Murdoch University, Murdoch, WA 6150, Australia; y.ren@murdoch.edu.au; 5 Agricultural Genomics Institute at Shenzhen, Chinese Academy of Agricultural Sciences, Shenzhen 510642, China

**Keywords:** *Bactrocera dorsalis*, insect gut microbiota, invasive insects, food source adaptation, gut microbiota function

## Abstract

**Simple Summary:**

The colonization of new areas by invasive insects has been a prominent concern in entomology. *Bactrocera dorsalis* serves as a typical invasive insect, which has expanded its range from southern to northern regions of China in recent years. Host suitability plays a crucial role in the successful establishment of *B. dorsalis* populations. In this study, we have observed a decline in the fitness of *B. dorsalis* when feeding on cucumber, primarily due to significant changes in the gut microbiota composition of the insect. Among them, *Empedobacter brevis* and *Enterococcus faecalis* were identified as key factors leading to the reduced fitness of *B. dorsalis*. These findings confirm the close association between insect fitness and symbiotic bacteria within the insect’s body. Furthermore, the source of food and gut bacteria have significant impacts on the invasion of exotic insects.

**Abstract:**

How alien pests invade new areas has always been a hot topic in invasion biology. The spread of the *Bactrocera dorsalis* from southern to northern China involved changes in food sources. In this paper, in controlled conditions, we take *Bactrocera dorsalis* as an example to study how plant host transformation affects gut bacteria by feeding it its favorite host oranges in the south, its favorite host peaches and apples in the north, and feeding it cucumbers as a non-favorite host plant, thereby further affecting their fitness during invasion. The result showed that, after three generations of feeding on cucumbers, *Bactrocera dorsalis* took longer to develop as a larva while its longevity and fecundity decreased and pre-adult mortality increased. Feeding it cucumbers significantly reduced the overall diversity of gut microbiota of *Bactrocera dorsalis*. The relative abundance of *Enterobacter* necessary for survival decreased, while the *Empedobacter* and *Enterococcus* increased, resulting in decreased carbohydrate transport and metabolism and increased lipid transport and metabolism. Feeding *Bactrocera dorsalis Empedobacter brevis* and *Enterococcus faecalis* resulted in a 26% increase in pre-adult mortality and a 2–3 d increase in adult preoviposition period (APOP). Additionally, *Enterococcus faecalis* decreased the longevity of female and male adults by 17 and 12 d, respectively, and decreased fecundity by 11%. We inferred that the shifted plant hosts played an important role in posing serious harm to *Bactrocera dorsalis* invading from the south to the north. Therefore, after an invasion of *Bactrocera dorsalis* into northern China, it is difficult to colonize cucumbers for a long time, but there is still a risk of short-term harm. The findings of this study have established that the interactions between an insect’s food source and gut bacteria may have an important effect on insect invasions.

## 1. Introduction

Economic globalization, climate change, and the flourishing transportation industry have triggered an upsurge in biological invasion [1], which has become an important ecological and environmental problem facing the world [2]. Since 2000, there have been at least 5–6 new invasive alien species found in China each year, and the transmission rate shows a significant annual growth [3]. So far, over 660 invasive alien species have been discovered in China, posing a serious threat to the ecological environment and agricultural security. Various major invasive pests, including *Bemisia tabaci* (Gennadius) (Homoptera: Aleyrodidae) [4], *Bactrocera dorsalis* (Hendel) (Diptera: Tephritidae) [5], *Solenopsis invicta* (Buren) (Hymenoptera: Formicidae) [6], *Spodoptera frugiperda* (Smith) (Lepidoptera: Noctuidae) [7], and *Cydia pomonella* (Linnaeus) (Lepidoptera: Tortricidae) [8], have successfully invaded China, and their rampancy areas have been expanding year after year [4,5,6,7,8].

Multiple hypotheses have been proposed in exploring biological invasion, such as the evolution of increased competitive ability [9], the enemy release hypothesis [10], and the mutualistic facilitation hypothesis [11]. These hypotheses explain the mechanisms of biological invasion from different perspectives, but many scholars hold that a large number of microorganisms present in the insect’s outer external cuticle, gut, or special cells play a crucial role in stress resistance, reproduction, digestion, and ecological adaptability [12,13]. This suggests that the microorganisms in insect bodies may promote insect adaptation and accelerate their invasion process through plant–insect coevolution [14]. However, it is still unclear how host food regulates the microorganisms in insect bodies, thereby affecting the invasion of alien insects [15].

Successful invasion of alien insects generally consists of four stages, including introduction, colonization, incubation, and dispersal, and the adaptation to new host foods is the first barrier they face after introduction [16]. Studies have shown that host food is an important factor affecting the survival, growth, and reproduction of insects [17,18,19]. *B. dorsalis* that fed on pomegranates showed the fastest growth rate and strongest reproductive ability [17], but abnormal egg hatching and reduced larval survival occurred when feeding on olives and mangoes. This is supposed to be caused by the significant impact of phenol in these foods on the growth and development of *B. dorsalis* larvae [18,19].

Meanwhile, the host food of insects is closely related to the changes in their gut microbiota. Yang et al. [20] reported that the diversity of gut microbiota in *Plutella xylostella* (Lepidoptera: Plutellidae) significantly decreased after transferring from radish to peas for 17 generations. The diversity of gut microbiota in *Grapholita molesta* (Lepidoptera: Tortricidae) is highest when feeding on plums and lowest when feeding on apples [21]. In addition, gut microbiota is involved in regulating various physiological activities of host insects, such as regulating the development and metabolic homeostasis [22], enhancing pest resistance [23], and supplementing nutrition [24]. Therefore, for the invasive alien pests, adaptation to the host food in invasive areas is the first barrier they face [20,21]. Further research is needed on how the host food regulates gut bacteria, which in turn affects the growth, development, and reproduction of alien insects, thereby determining their survival and successful colonization of the local area [15].

*B. dorsalis* is a typical invasive pest known as a fruit and vegetable killer, mainly damaging citrus, carambola, and guava in southern China, seriously affecting the safe production of fruits [25]. Under the influence of global warming and frequent agricultural trade, its invasion range has gradually expanded [26]. In the 21st century, it has been found to have spread to northern regions such as Shaanxi, Henan [27], Beijing, and Hebei [28], and food hosts have also shifted from oranges, carambola, and guava to peaches, apples, and jujubes [29], causing serious harm to fruits in the north [28]. *B. dorsalis*, also known as a fruit and vegetable pest, has a damage rate of 27.7% to cucumbers in southern China [30], but no damage to cucumbers in the north has been found or reported, where they are widely planted and are one of the main vegetables.

Considering that *B. dorsalis* has invaded northern China and caused serious harm to peaches and apples in the region, this study uses *B. dorsalis* as an example by feeding it its favorite host oranges in the south, its favorite host peaches and apples in the north, and its non-favorite host cucumbers to study their effects on the growth, development, reproduction, and survival of *B. dorsalis*. The 16S rDNA sequencing is used to determine the diversity of the gut microbiota of *B. dorsalis* after feeding on different hosts. By isolating and cultivating differential bacteria, the biological function of gut microbiota on *B. dorsalis* is tested. This study is supposed to clarify the impact of the host food and gut microbiota on the growth, reproduction, and survival of *B. dorsalis* during its invasion from the south to the north and elucidate their important regulatory role in the successful invasion of alien pests into new areas, with the goal of enriching the invasion theory of invasive species.

## 2. Materials and Methods

### 2.1. Insects and Food Source Fruits

The population of *B. dorsalis* was obtained from the Innovative Team of Plant Quarantine and Alien Invasive Pest Control of South China Agricultural University. They were reared at 26 ± 1 °C, 65 ± 5%, with a photoperiod of 16:8 (L:D), according to the method for 2 years [31] (larval diet contained 150 g corn flour, 0.6 g sodium benzoate, 30 g yeast, 30 g sucrose, 30 g paper towel, 1.2 mL hydrochloric acid, and 300 mL water; adult diet consisted of water, yeast hydrolysate, and sugar). *B. dorsalis* was reared on apples, oranges, peaches, and cucumbers for two generations. Four different foods were purchased from Chengyang wholesale market in Qingdao, China. They were fresh, disease-free, and met national pesticide residue detection standards. Fruits were soaked in tap water for 2 h before use. For the function of gut microbiota experiments, *B. dorsalis* larvae were fed with an artificial diet consisting of a mixture of 150 g corn flour, 30 g yeast extract powder, 30 g sucrose, 30 g toilet paper, 1.2 mL concentrated hydrochloric acid, and 300 mL sterile water. The ingredients of the artificial diet were mixed and autoclaved at 121 °C for 20 min, cooled, and stored at 4 °C until use [32].

### 2.2. Development, Longevity, and Reproduction of B. dorsalis on Four Food Source Fruits

*B. dorsalis* were placed in a plastic larva box (35 mm in diameter) with 5 g of fruit, and hatched larvae were transferred individually to a new larva box with a brush. The developmental duration, pre-adult mortality, APOP (adult preoviposition period), and reproductive value (*v_xj_*) of each insect were measured and analyzed following Zhu et al. [33]. The four food source experiments all have three replicates. Each replicate contains 30 insects, totaling 90 insects.

### 2.3. Host Preference of B. dorsalis on Four Food Source Fruits

Thirty females and 30 males at 25 d after emergence were introduced into a rearing cage (37 cm × 37 cm × 37 cm). Four corners of the cage were each provided with a fruit (the same size) as feeding, puncturing, and oviposition sites for *B. dorsalis*. The observation time was from 7:00 to 17:00, and the number of visits and oviposition punctures of *B. dorsalis* on the fruit were recorded. After 72 h, the fruit was dissected, and the number of larvae in the fruit was recorded. The experiment was repeated 20 times.

### 2.4. Diversity of Microbiota from B. dorsalis Larvae Gut

Five-day-old larvae were selected from fruit samples and soaked in 75% ethanol for 5 s washed with sterile water 3 times. The soaked larvae were dissected under a stereomicroscope. For each sample, 50 midguts were dissected, and 3 samples were collected. Total DNA of the dissected gut samples was extracted using a DNA extraction kit (Tiangen, Beijing, China) following the manufacturer’s instructions. To assess DNA quality, a 1.2% agarose gel was used. The 16S rDNA V3-V4 hypervariable region was amplified according to the protocol provided in Text A1. The sequencing library was prepared using the Illumina Hiseq 2500 (Biomarker Co., Ltd., Beijing, China), and the data were uploaded to sequence read archive accession number PRJNA1010131.

The data were spliced after quality control and divided into multiple OTUs (Operational Taxonomic Units) with 97% sequence similarity and then annotated based on the SILVA database. One-way ANOVA was used to compare the results of the alpha diversity index (Shannon, ACE, Chao) and abundance of gut bacteria of *B. dorsalis* on four host fruits. Finally, the key bacterial function was predicted according to the COG database. The data were analyzed using Majorbio Cloud Platform (www.majorbio.com, accessed on 1 December 2023).

The raw data were processed by Fastp (Version 0.19.6, https://github.com/OpenGene/fastp, accessed on 1 November 2023) quality control and filtered sequencing, and the high-quality reads were spliced according to the overlap relationship. With 97% sequence similarity, Usearch (Version 10.0, http://www.drive5.com/usearch/, accessed on 1 November 2023) was divided into multiple OTUs, and then representative sequences were annotated and analyzed with SILVA database (version 138, https://www.arb-silva.de/, accessed on 1 November 2023). Principal coordinate analysis (PCoA) was used to reveal the differences in bacterial communities in the gut of *B. dorsalis,* and one-way ANOVA was used to compare the significant differences. Finally, PICRUSt 2 (Version 2.2.0, https://github.com/picrust/picrust2/, accessed on 1 November 2023) was used to annotate the OTUs pathway according to the COG database to predict the differential bacterial function.

### 2.5. Gut Bacteria Isolation and Culture

Based on the previous life table and 16S rDNA experiments, six 5-day-old *B. dorsalis* larvae that fed on cucumbers were collected and immediately soaked in 75% ethanol for 5 s. The guts of *B. dorsalis* were dissected and collected in sterile centrifuge tubes to which 50 μL of sterile water was added. The guts were then ground with sterile grinding pestles, and the fluid was streaked and cultivated for 48 h at 30 °C on mrs broth (MRS) and nutrient (NA) agar flat plates (Text A2). Colonies with the same morphology were selected for subculturing. The pure cultures were inoculated into MRS and NA medium, and the liquid cultures were stored in 25% glycerol solution under −80 °C.

16S rDNA of the cultivated bacteria was amplified and sequenced (Text A1). The sequences were subjected to a BLAST search against the NCBI database for sequence homology analysis. The GenBank accession numbers for *E. faecalis* (accession number: 2739092) and *E. brevis* (accession number: 2739079). According to the sequencing results, a phylogenetic tree was constructed using the neighbor-joining method.

### 2.6. Functions of E. brevis and E. faecalis

One mL of 1 × 10^8^ cfu/mL *Empedobacter brevis* or *Enterococcus faecalis* bacterial solution (CK was PBS buffer) (Text A3) was added to a larva feeding box containing 3 g of artificial diet and, after mixing, 1 newly hatched larva was added. Growth and development of the larva were observed daily. Each assay consisted of 90 replicates.

### 2.7. Statistical Analysis

Life table data were processed by TWOSEX-MSChart software (Version 2024.01.06, accessed on 1 February 2024) based on the theory of two sex life tables [34]. The mean and standard errors of life table parameters (*r*, *λ*, *R*_0_, *T*), pre-adult duration, longevity, and mortality were calculated by bootstrap with 100,000 resamplings. The confidence intervals of the paired bootstrap tests were used to detect the difference between the treatments [35,36]. TIMING-MSChart (Version 2024.01.06, accessed on 1 February 2024) predicts the number of individuals in the population. A Chi-square test was used to analyze the number of visits, oviposition punctures, and larval numbers of *B. dorsalis* on the fruits. Finally, the graphs were plotted using SigmaPlot (Version 12.0, accessed on 1 February 2024).

## 3. Results

### 3.1. Development, Longevity, and Reproduction of B. dorsalis on Four Food Sources

Larvae had an extended pre-adult development period and shortened longevity when feeding on cucumbers compared to oranges, peaches, and apples. The larval duration and pupal duration of *B. dorsalis* that fed on cucumbers had the longest pre-adult development periods of all the food sources tested, significantly longer than those achieved with oranges (larvae, *p* < 0.001; pupae, *p* < 0.001) or peaches (larvae, *p* < 0.001; pupae, *p* = 0.036). The longevity of adult *B. dorsalis* that fed on cucumbers was significantly shorter than when fed oranges (*p* < 0.001, *p* < 0.001), peaches (*p* < 0.001, *p* < 0.001), or apples (*p* < 0.001, *p* < 0.001). Pre-adult mortality of the *B. dorsalis* that fed on cucumbers (77%) was significantly greater than those that fed on oranges (*p* < 0.001), peaches (*p* < 0.001), and apples (*p* < 0.001) by 46%, 48%, and 44%, respectively (Table 1).

**Figure 1:**
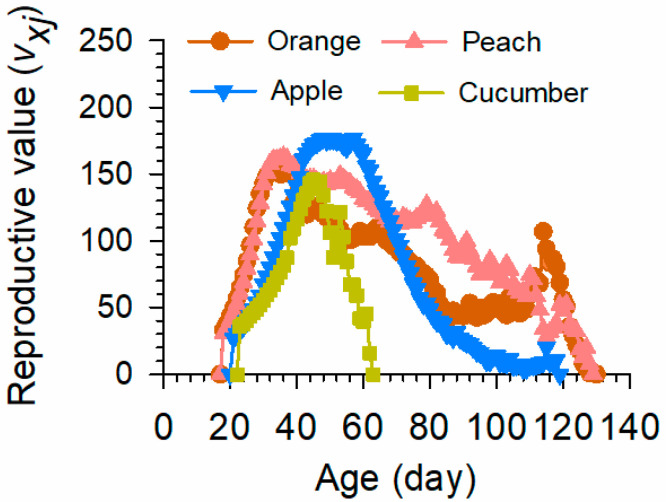
Reproductive value (*v_xj_*) of *B. dorsalis* on four food source fruits. *v_xj_* is the contribution of individuals at age *x* and stage *j* to the future population.

The APOP of *B. dorsalis* that fed on cucumbers was significantly longer than that of those that fed on oranges (*p* < 0.001) and peaches (*p* < 0.001) (Table 1). The reproductive value of *B. dorsalis* that fed on cucumbers was 3350 at 39 d, significantly less than of those that fed on oranges (9050 at 111 d), peaches (11,061 at 110 d), and apples (7549 at 97 d) (Figure 1).

### 3.2. B. dorsalis Shows a Preference for Oranges and Peaches in Northern China Rather Than Cucumbers

**Figure 2:**
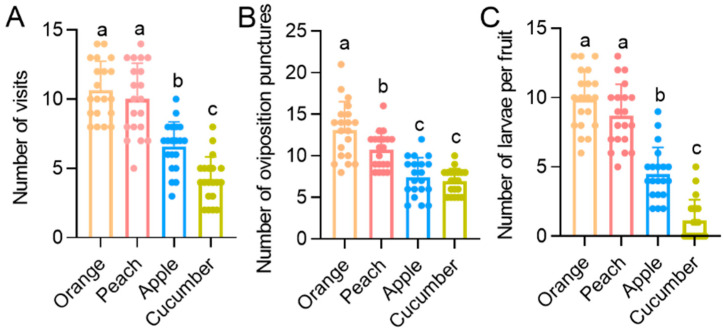
The number of visits (**A**), number of oviposition punctures (**B**), and number of larvae per fruit (**C**) of *B. dorsalis* on four food source fruits. Selection preference was analyzed using Chi-squared test. Data are expressed as mean ± SD, and different letters indicate significant differences between treatments.

Our cage experiment is also consistent with the life table results. The number of visits closely related to *B. dorsalis* feeding is only 4.2 times on cucumbers, which is significantly less than on oranges (10.6, *p* < 0.001), peaches (10, *p* < 0.001), and apples (6.6, *p* = 0.002) (Figure 2A). The number of oviposition punctures on cucumbers (6.9) also significantly decreased, significantly less than on oranges (13.1, *p* < 0.001) and peaches (10.7, *p* < 0.001) (Figure 2B). Moreover, the number of larvae in cucumbers (1.2) was significantly less than in oranges (9.9, *p* < 0.001), peaches (8.7, *p* < 0.001), and apples (4.5, *p* < 0.001) (Figure 2C).

### 3.3. Feeding on Different Hosts Results in Different Gut Microbiota Communities

**Figure A1:**
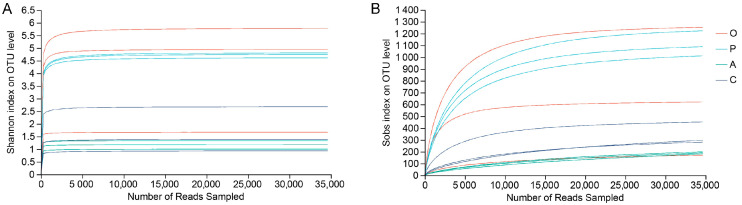
The Sobs index (**A**) and Shannon index (**B**) rarefaction curves. O, orange-feeding; P, peach-feeding; A, apple-feeding; C, cucumber-feeding.

Sequencing yielded a total of 531,326 sequences, with an average of 44,277 sequences per sample. The optimized sequences comprised 225,068,777 bases, with an average sequence length of 423 base pairs (Table A1). Rarefaction curves demonstrated that sequencing depth was sufficient, and most bacteria in the samples were identified (Figure A1).

The alpha diversity index analysis revealed differences in the gut microbiota of *B. dorsalis* that fed on different food sources (Table 2). The Shannon diversity index was greatest in larvae feeding on peaches, significantly greater than for those feeding on apples (*p* < 0.001) and cucumbers (*p* = 0.004); the ACE and Chao indices also showed that the diversity of bacteria was significantly greater in those feeding on peaches compared to those feeding on apples (*p* < 0.001, *p* < 0.001) and cucumbers (*p* < 0.001, *p* < 0.001).

**Figure 3:**
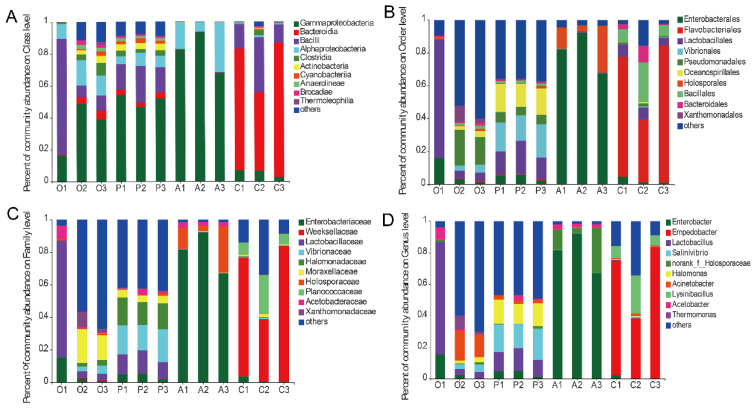
Microbiota composition of the top 10 relative abundances at class level (**A**), order level (**B**), family level (**C**), and genus level (**D**). Each color represents a species, and the height of the color block indicates the proportion of the species in relative abundance. Other species are incorporated as “Others”, as shown in the diagram. Food source fruits: O, orange; P, peach; A, apple; C, cucumber.

The gut microbiota composition of *B. dorsalis* that fed on different host fruits exhibits significant differences. It is primarily composed of Gammaproteobacteria, Bacteroidia, Bacilli, and Alphaproteobacteria, which collectively account for more than 90% of bacteria. Specifically, the predominant bacteria in the gut of *B. dorsalis* that fed on oranges (34.5%), peaches (50.9%), and apples (81.2%) belong to the Gammaproteobacteria, while the predominant bacteria for those that fed on cucumbers are Bacteroidia (Figure 3A). In the guts of *B. dorsalis* larvae that fed on apples, the taxa of greatest relative abundance were Enterobacteriaceae (80.2%) and *Enterobacter* (80.1%). For those that fed on cucumbers, the dominant taxa were *Flavobacteriales* (64.8%), *Weeksellaceae* (64.8%), and *Empedobacter* (64.7%). In the guts of larvae that fed on oranges and peaches, the dominant category was “others”, indicating greater diversity in the gut bacteria than of those that fed on apples and cucumbers (Figure 3B–D).

**Figure 4:**
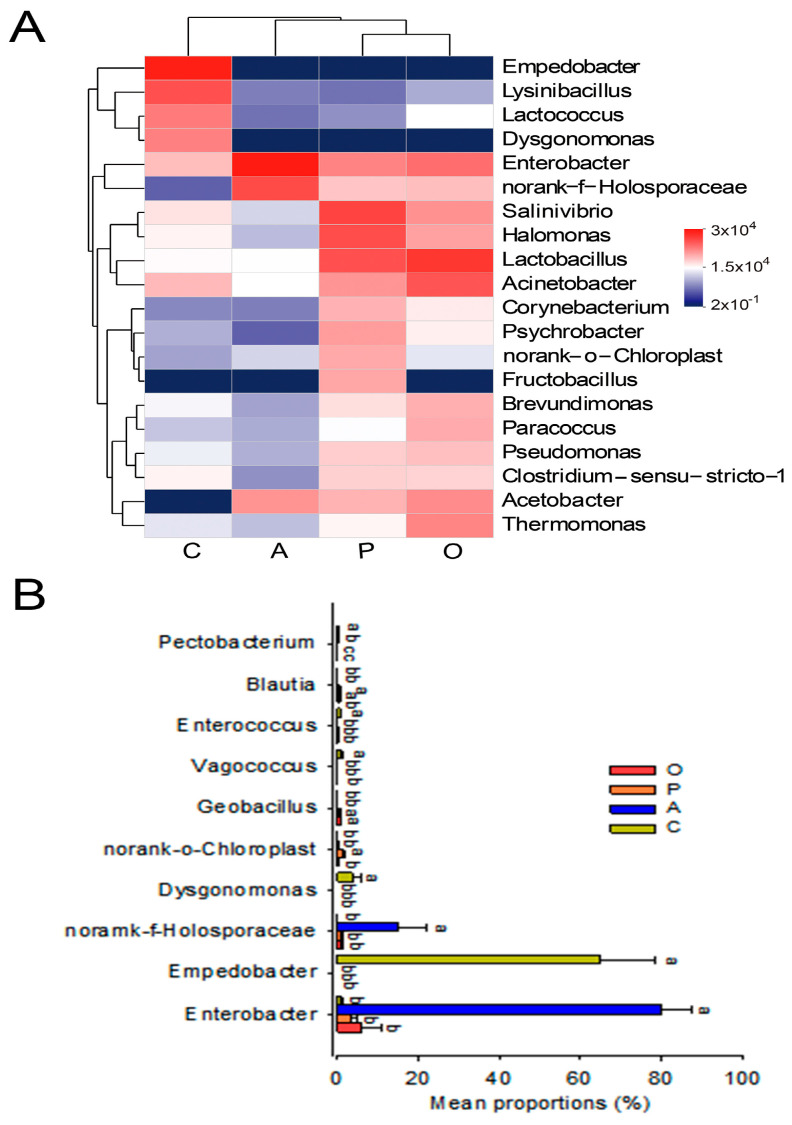
Cluster heatmap of the Top20 genera at the genus level (**A**) and bar plot of the significantly different Top10 genera (**B**). Food source fruits: O, orange; P, peach; A, apple; C, cucumber. Different lowercase letters on columns indicate significant differences (one-way ANOVA, Tukey post hoc test, *p* < 0.05).

**Figure A2:**
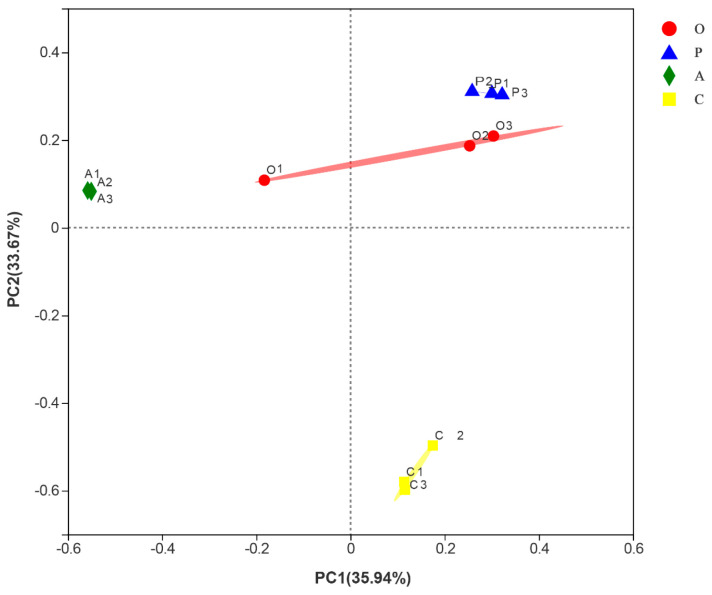
Principal coordinate analysis (PCoA) based on the Bray–Curtis distance between different host diets. O, orange-feeding; P, peach-feeding; A, apple-feeding; C, cucumber-feeding.

Based on the clustering heat map, we found that samples feeding on oranges and peaches clustered together, those feeding on apples clustered on adjacent branches, and those feeding on cucumbers formed a separate cluster. This suggests the most similarity in gut microbiota between *B. dorsalis* that fed on oranges and peaches, followed by those that fed on apples, while the gut microbiota of those that fed on cucumbers was distinct (Figure 4A), consistent with the PCoA analysis (Figure A2). Notably, the relative abundance of *Empedobacter* (orange: <0.01%, peach: 0.00%, apple: 0.24%, cucumber: 64.53%), *Lysinibacillus* (0.03%, 0.01%, 0.01%, 12.66%) and *Dysgonomonas* (0.00%, 0.00%, 0.00%, 3.86%) was increased in the gut of *B. dorsalis* that fed on cucumbers. By analyzing the differential bacteria, it was found that *Empedobacter* (64.53%), *Dysgonomonas* (3.86%), *Vagococcus* (1.08%), and *Enterococcus* (0.85%) increased their relative abundance in the gut of *B. dorsalis* that fed on cucumbers (Figure 4B).

### 3.4. Prediction of Key Gut Bacteria Functions

**Figure 5:**
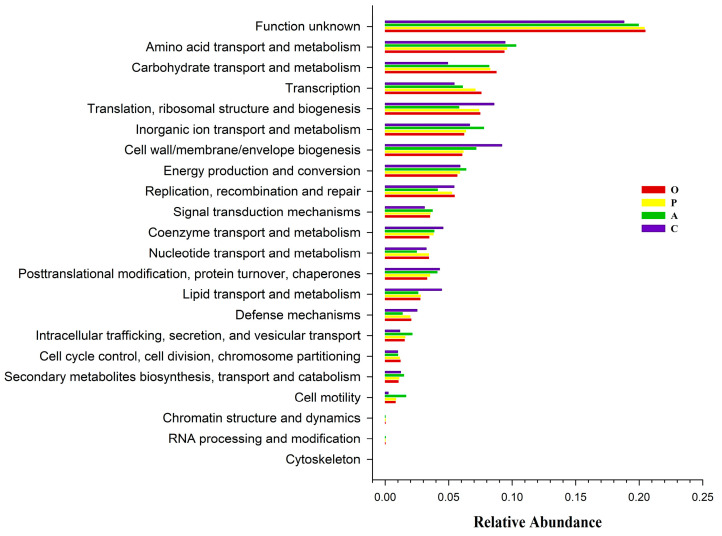
Comparison of predicted COG functions of gut bacteria of B. dorsalis that fed on different food sources. Food source fruits: O, orange; P, peach; A, apple; C, cucumber.

**Figure A3:**
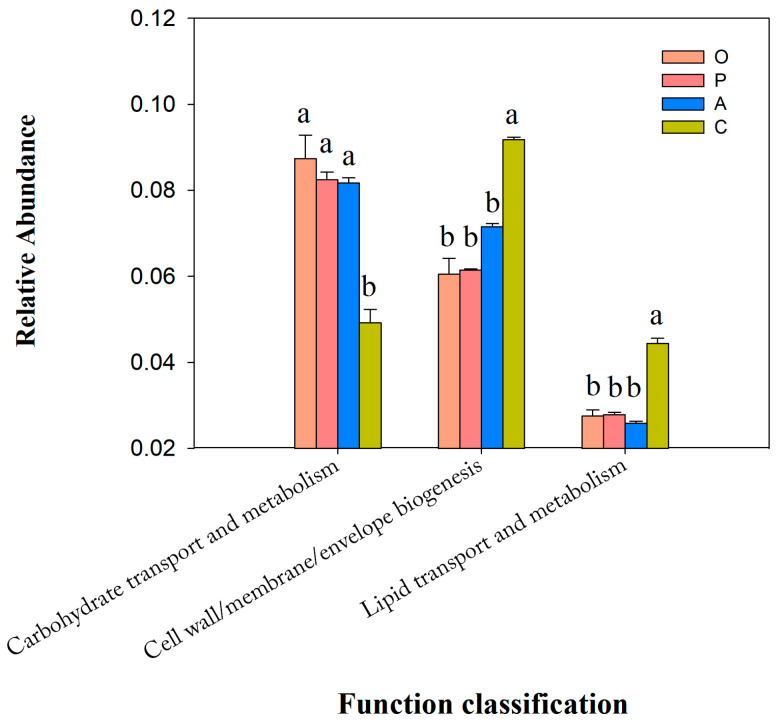
Significant differences in functional abundance of carbohydrate transport and metabolism, cell wall/membrane/envelope biogenesis, lipid transport, and metabolism. Different lowercase letters on columns indicate significant differences (one-way ANOVA, Tukey post hoc test, *p* < 0.05).

PICRUSt2 software (Version 2.2.0, https://github.com/picrust/picrust2/, accessed on 1 November 2023) was used to predict functions of the microbiota detected in *B. dorsalis* gut on different food sources. The results predicted that these bacteria were mainly associated with energy transport and metabolic functions. Notably, the relative abundance of bacteria associated with carbohydrate transport and metabolism in the midguts of *B. dorsalis* that fed on cucumbers was reduced by approximately 30% compared to those that fed on the other fruits, while bacteria associated with cell wall/membrane/envelope biogenesis and lipid transport and metabolism were more abundant by approximately 25% and 17%, respectively (Figure 5). This suggests that *B. dorsalis* obtains significantly fewer nutrients and energy resources when feeding on cucumbers, while the demand for substances involved in membrane formation and lipid metabolism increases (Figure A3).

### 3.5. E. brevis and E. faecalis Affect the Development, Longevity, and Reproduction of B. dorsalis

**Figure A4:**
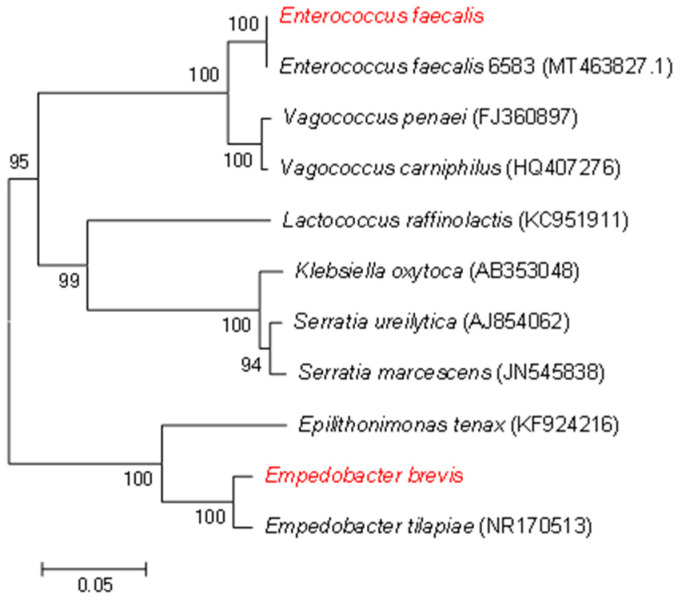
The phylogenetic tree based on 16S rDNA sequences, constructed by the neighbor-joining method, showing the relationship between strain and representatives of some related taxa. Numbers in parentheses are accession numbers of published sequences. Bootstrap values (3000 replications) are shown as percentages at branch points. The strains we obtained are marked in red.

*E. faecalis* was isolated on the MRS medium, and *E. brevis* was isolated on the NA medium (Figure A4). Feeding on these two gut bacteria affected the development, longevity, and reproduction of *B. dorsalis*. Notably, pre-adult mortality (*p* = 0.005, *p* = 0.004) significantly increased compared to the CK. The APOP was significantly longer (*E. brevis*, *p* < 0.001; *E. faecalis*, *p* = 0.001;). After being fed *E. faecalis*, female and male adult longevity was significantly shorter than that of the CK group (female *p* < 0.001, male *p* = 0.005) and the *E. brevis* group (female *p* = 0.001, male *p* = 0.037) (Table 3).

Feeding *B. dorsalis E. faecalis* significantly reduced fecundity compared to CK (*p* = 0.012) and those fed *E. brevis* (*p* = 0.008). The number of oviposition days was not significantly different amongst the groups. Life table parameters showed that *B. dorsalis* fed *E. faecalis* had a reduced *R*_0_ (*p* = 0.008, *E. brevis*: *p* = 0.006) and shortened *T* (CK: *p* < 0.001, *E. brevis*: *p* = 0.006), while *r* and *λ* showed no significant differences (Table 3).

**Figure A5:**
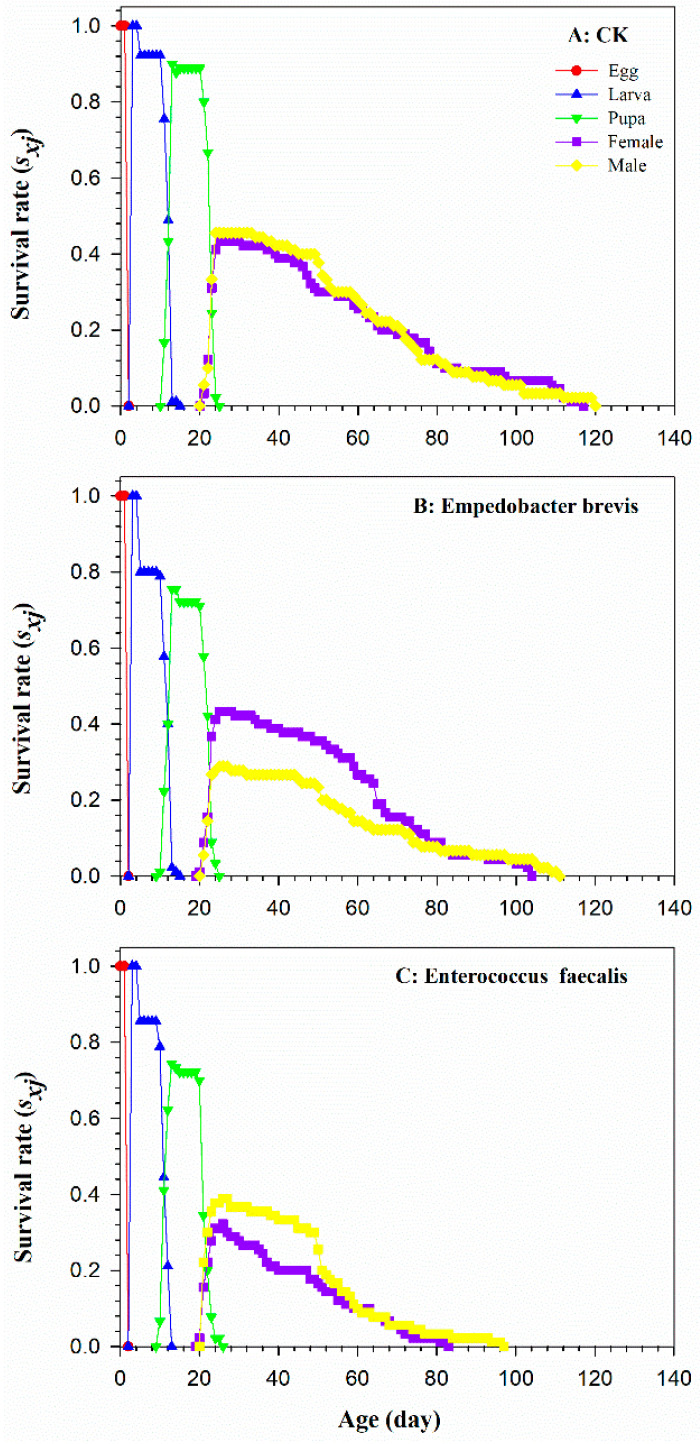
Age-stage specific survival rate (*s_xj_*) of *B. dorsalis* fed different gut microbiota.

**Figure A6:**
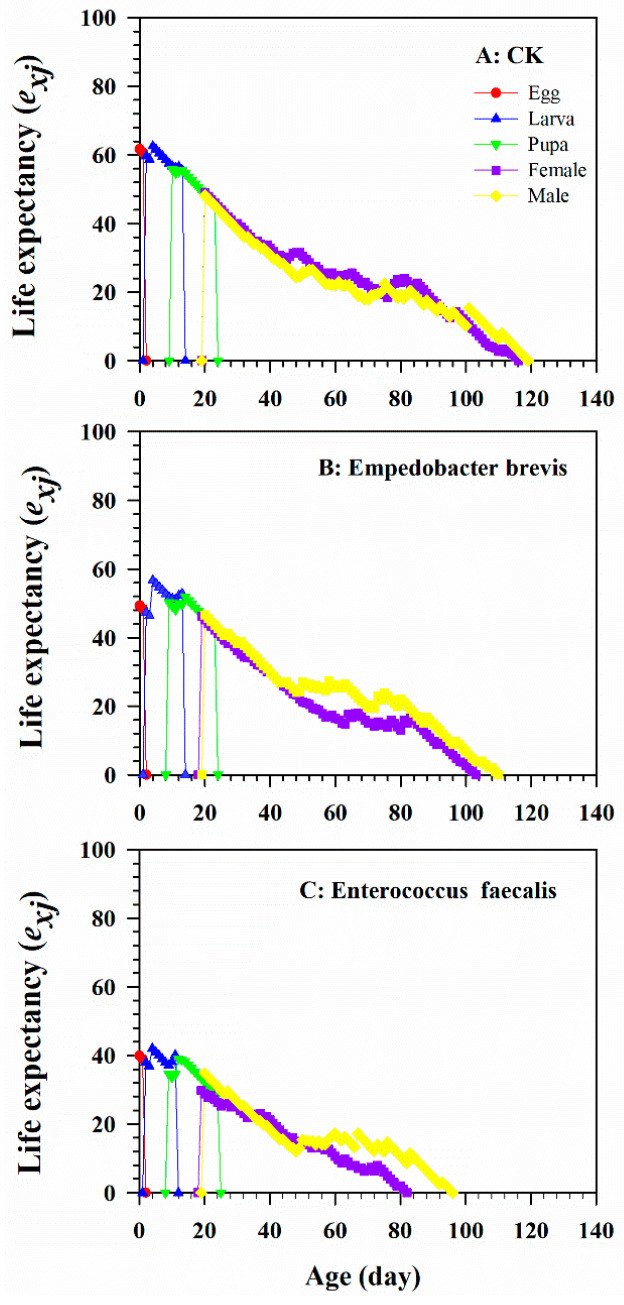
Age-stage specific life expectancy (*e_xj_*) of *B. dorsalis* fed different gut microbiota.

**Figure A7:**
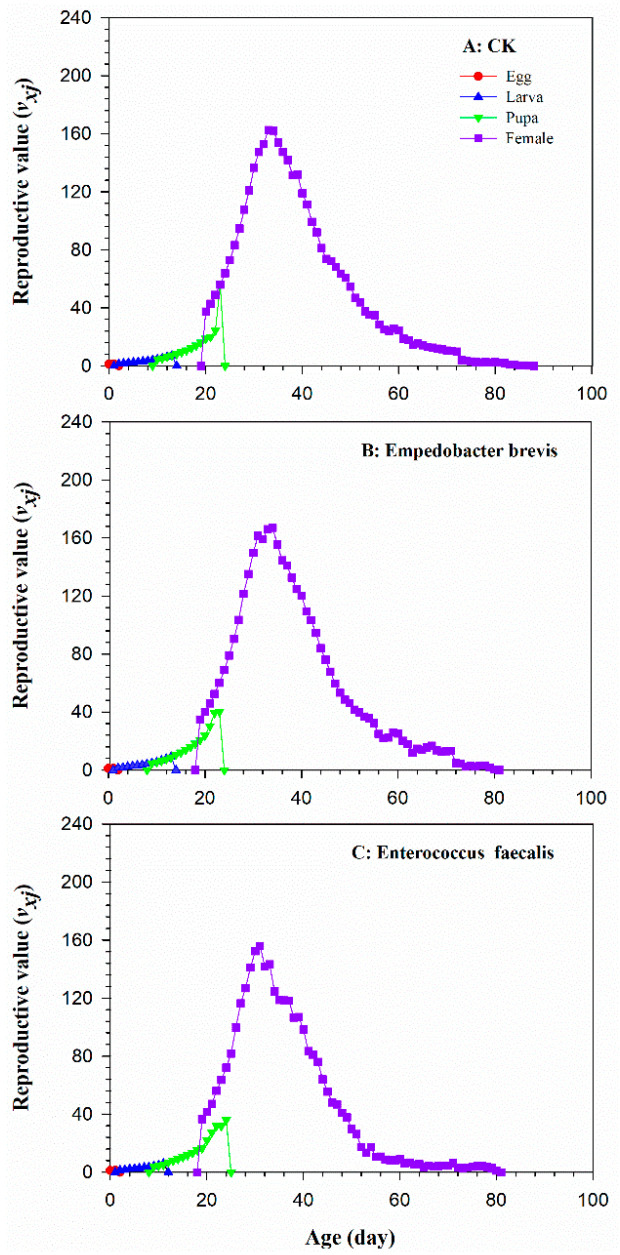
Reproductive value (*v_xj_*) of *B. dorsalis* fed different gut microbiota.

Feeding on both bacterial species decreased survival rates in *B. dorsalis* larvae (CK: 92.2%, *E. brevis*: 80.0%, *E. faecalis*: 85.6%) and pupae (CK: 88.9%, *E. brevis*: 72.2%, *E. faecalis*: 72.2%) (Figure A5), which contributed to the increased pre-adult mortality (Table 3). At age 0 (*e*_01_), *B. dorsalis* fed *E. faecalis* had the shortest life expectancy (39.9 d) compared to those fed *E. brevis* (49.3 d) and the CK group (61.7 d) (Figure A6). The fecundity production time of *B. dorsalis* fed with gut bacteria (*E. brevis*: 61.0 d, *E. faecalis*: 61.0 d) was shorter than that of CK (67.0 d) (Figure A7).

**Figure A8:**
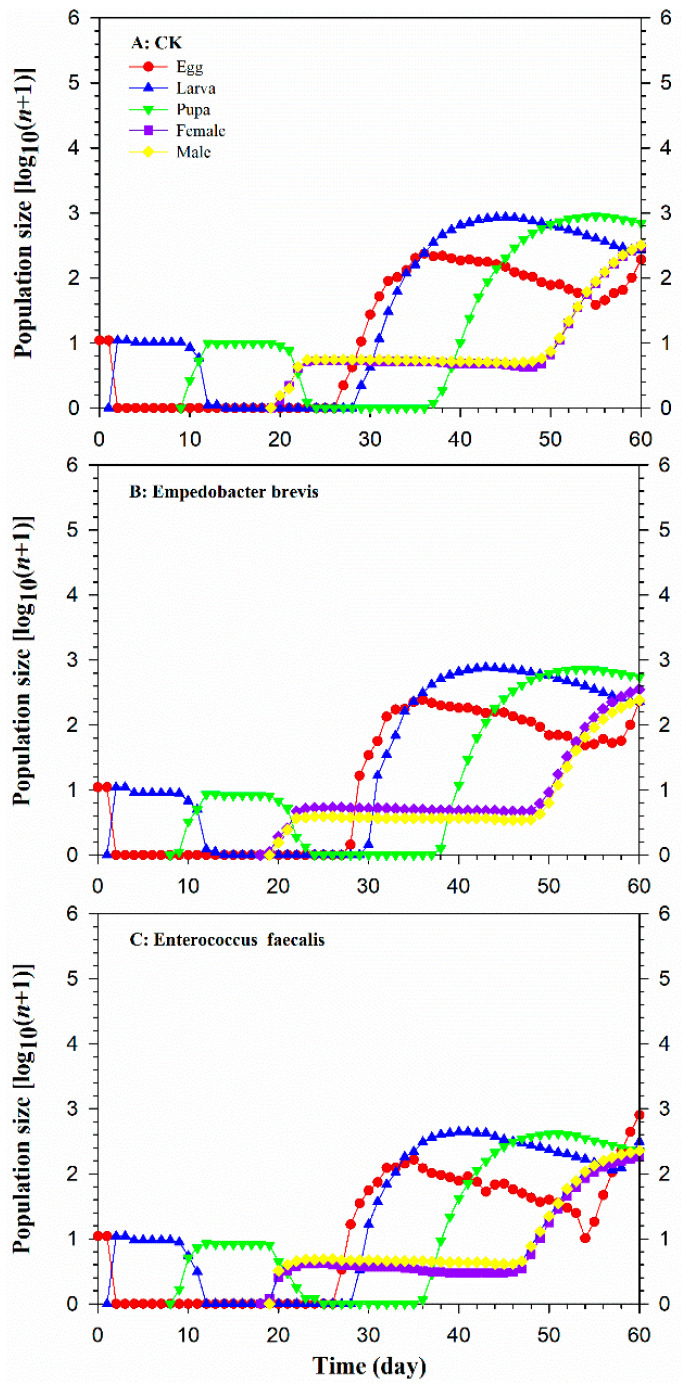
Population projection of *B. dorsalis* fed different gut microbiota.

Using the experimental data in the TIMING-MSChart program to predict population size from an initial 10 eggs, the populations of *B. dorsalis* fed with gut microbiota showed a significant reduction in adult numbers after 60 d (*E. brevis*: 594, *E. faecalis*: 404) compared to the CK population (624) (Figure A8).

## 4. Discussion

### 4.1. Food Source Affects the Development, Longevity, Reproduction, and Host Preference of Insects

The kind and quality of food sources are closely related to the survival, growth, development, and reproduction of herbivorous insects [17,18,19,35,36,37]. This experiment studied the development, longevity, and reproduction of *B. dorsalis* after its transition to fruits and cucumbers extensively grown in northern China. Peaches and apples were suitable for *B. dorsalis* development and reproduction, while cucumbers were not. The developmental duration and APOP increased, adult longevity and fecundity production time decreased, and the pre-adult mortality rate increased when *B. dorsalis* were fed cucumbers. In summary, after invading northern regions, the fitness of *B. dorsalis* that fed on cucumbers was very low. If feeding on cucumbers continuously for multiple generations, it will face difficulties in completing its growth and development. But based on *B. dorsalis,* it can be completed in three successive generations of cucumber life history; therefore, we hypothesize that when the northern lacks, the host of the worm on the cucumber is still at risk of harm, so the surveillance should be strengthened.

It has been reported that excessive phenolic and latex in unsuitable food sources can lead to an imbalance of gut homeostasis, thereby affecting the survival of insects [19,38]. Which secondary metabolites in cucumbers are related to bacterial homeostasis in the gut deserves further study when the food source was shifted to cucumbers.

### 4.2. Host Source Affects Gut Bacterial Diversity of Insects

Gut bacteria often participate in the food source’s carbohydrate metabolism [39] and other life activities, playing an important role in the adaptation [40] and invasion [15] of insects. Therefore, this study investigated the shift in the gut microbiota of *B. dorsalis* caused by food source transformation. Alpha diversity comparison, cluster heat maps, and PCoA showed that the diversity was similar in individuals feeding on oranges and peaches, followed by those feeding on apples. In contrast, individuals feeding on cucumbers had significantly decreased diversity of gut microbiota. Similar results have been found in Lepidoptera. For example, *P. xylostella* showed a significant decrease in gut microbiota diversity after transitioning from its preferred food source, radish, to a non-preferred food source, pea [20].

Proteobacteria in insect intestines have an important impact on insect adaptation to specific food source plants [20]. This study showed the dominant bacteria in the gut of *B. dorsalis* that fed on oranges, peaches, and apples were Gammaproteobacteria, while Bacteroidia were dominant in individuals feeding on cucumbers. This may explain the overall reduced fitness of the insects fed with cucumbers. Further analysis revealed a significant increase in *Empedobacter*, *Dysgonomonas*, *Vagococcus*, and *Enterococcus* in the gut of cucumber-fed *B. dorsalis*. *Empedobacter* is known to proliferate abundantly within insects and produce proteinaceous toxins, commonly used as a biopesticide for controlling Lepidopteran insects such as *S. frugiperda* [41]. In bat research, it has been confirmed that *Vagococcus* can be associated with 86 virulence factors, leading to abnormal cellular adhesion processes [42]. Li et al. [43] reported that *Henosepilachna vigintioctopunctata* (Fabricius) (Coleoptera: Coccinellidae) ceases to oviposit in the presence of *Enterococcus* in its gut. Studies by Akami et al. [44] and Noman et al. [45] have also shown that *Enterococcus* has inhibitory effects on the reproduction of *B. dorsalis* and *Zeugodacus tau* (Walker) (Diptera: Tephritidae).

Analysis of differential gut microbiota of *B. dorsalis* found that they were mainly related to metabolism and cellular processes. Notably, *B. dorsalis* that fed on cucumbers showed a significant decrease in gut Gammaproteobacteria and the processes of carbohydrate transport and metabolism in which these bacteria have an important role [20]. This process involved nutrient uptake and energy production, which were closely associated with the growth, development, and reproduction of the insect.

The prediction of gut microbiota function also found that *B. dorsalis* that fed on cucumbers showed a significant increase in lipid transport and metabolism. Lipids have been closely associated with insect reproduction [46] as they were essential for synthesizing vitellogenin [47], influencing the quantity and quality of eggs [48], and providing nutrition for embryonic development [49]. Also, lipids can directly and indirectly affect the fecundity of insects [50]. Based on these findings, it can be inferred that after *B. dorsalis* transitioned to feeding on cucumbers, the relative abundance of gut microbiota (Gammaproteobacteria) decreased, and its capability for carbohydrate transport and metabolism was also reduced. To maintain its longevity and fecundity, *B. dorsalis* that fed on cucumber may have used its own lipids as an alternative energy source [51]. The abnormal survival, development, and fecundity of *B. dorsalis* feeding on cucumbers can be attributed to these physiological changes.

### 4.3. Key Differential Bacteria Regulate the Fitness of Invasive Alien Insects

When *B. dorsalis* larvae were fed isolated *E. brevis* and *E. faecalis*, both bacteria were detrimental to population growth, as evidenced by increased pre-adult mortality and extended APOP. This result concurs with previous findings where *E. brevis* was shown to reduce the survival rate of *S. frugiperda* [39], and *E. faecalis* decreased adult longevity and fecundity in *H. vigintioctopunctata* [41]. Additionally, studies on *B. dorsalis* [42] and *Z. tau* [43] have also demonstrated the inhibitory effect of *Enterococcus* on reproduction. These findings confirm that feeding on cucumbers changed the relative abundance of key bacteria such as *E. brevis* and *E. faecalis* within the *B. dorsalis* gut, which led to the disruption of their development and reproduction.

## 5. Conclusions

In this study, we found for the first time that cucumbers are not suitable for the growth and development of *B. dorsalis* in the process of transitioning from preferred food sources grown in southern China to alternative hosts grown in the north. The unsuitability of cucumbers as a food source was correlated to a significant reduction of bacterial diversity in the gut, while relative increases in unfavorable bacteria *E. brevis* and *E. faecalis* further reduced *B. dorsalis* fitness. Our findings help to understand the interactions between gut bacteria and insect invasions, offering new insights into microbial-mediated food source adaptability mechanisms and the potential for insect invasion management.

## Figures and Tables

**Table 1:** Mean (±SE) of larval duration, pupal duration, female/male adult longevity, pre-adult mortality, and adult preoviposition period (APOP) of *B. dorsalis* that fed on four food source fruits. Standard errors were estimated using 100,000 bootstrap resampling, and a paired bootstrap test was used to detect differences between treatments. Different lowercase letters in the same line indicate a significant difference (*p* < 0.05).

Statistics	Orange	Peach	Apple	Cucumber
Larval duration (d)	7.68 ± 0.08 d	8.09 ± 0.08 c	8.50 ± 0.08 b	9.04 ± 0.19 a
Pupal duration (d)	9.85 ± 0.06 c	10.14 ± 0.06 b	10.22 ± 0.09 ab	10.48 ± 0.15 a
Female adult longevity (d)	71.03 ± 3.23 a	71.81 ± 3.32 a	63.81 ± 4.30 a	29.73 ± 2.09 b
Male adult longevity (d)	62.71 ± 2.19 a	53.09 ± 2.21 b	57.26 ± 2.68 ab	22.80 ± 1.91 c
Pre-adult mortality (%)	31.11 ± 4.87 b	28.89 ± 4.78 b	33.33 ± 4.96 b	76.67 ± 4.45 a
Adult preoviposition period (APOP) (d)	9.85 ± 0.38 b	8.97 ± 0.22 c	19.17 ± 1.04 a	20.10 ± 0.62 a

**Table 2:** Mean (±SE) of Shannon, ACE, and Chao index of gut bacteria of *B. dorsalis* that fed on four food sources. Different letters indicate significant differences (one-way ANOVA, Tukey post hoc test, *p* < 0.05) in the mean values.

Parameters	Orange	Peach	Apple	Cucumber
Shannon index	4.13 ± 2.17 ab	4.73 ± 0.10 a	1.17 ± 0.17 b	1.67 ± 0.91 b
ACE index	699.31 ± 533.71 ab	1134.90 ± 103.88 a	295.72 ± 39.38 b	400.31 ± 66.48 b
Chao index	704.18 ± 532.92 ab	1143.60 ± 101.99 a	280.23 ± 32.37 b	401.36 ± 69.43 b

**Table 3:** Mean (±SE) of the pre-adult mortality, adult preoviposition period (APOP), female/male adult longevity, fecundity, oviposition days, intrinsic rate of increase (*r*), finite rate (*λ*), net reproductive rate (*R*_0_), and mean generation time (*T*) of two gut bacteria of *B. dorsalis*. Standard errors were estimated using 100,000 bootstrap resampling. The paired bootstrap test was used to detect the differences between treatments. Different letters indicate significant differences (*p* < 0.05).

Parameters	CK	*Empedobacter brevis*	*Enterococcus faecalis*
Pre-adult mortality (%)	11.11 ± 3.31 b	27.77 ± 4.71 a	27.80 ± 4.73 a
APOP (d)	7.26 ± 0.21 b	10.76 ± 0.63 a	9.23 ± 0.51 a
Female adult longevity (d)	47.02 ± 3.87 a	43.61 ± 3.07 a	28.31 ± 3.16 b
Male adult longevity (d)	46.27 ± 3.40 a	45.19 ± 4.62 a	34.03 ± 2.67 b
Fecundity (eggs per female)	428.39 ± 31.39 a	446.07 ± 37.52 a	292.60 ± 44.14 b
Oviposition days (d)	22.90 ± 1.70 a	20.76 ± 1.62 a	19.32 ± 2.07 a
*r* (d^−1^)	0.1328 ± 0.0041 a	0.1356 ± 0.0043 a	0.1258 ± 0.0064 a
*λ* (d^−1^)	1.1420 ± 0.0046 a	1.1455 ± 0.0050 a	1.1341 ± 0.0073 a
*R*_0_ (offspring/individual)	185.64 ± 26.06 a	193.36 ± 28.39 a	97.53 ± 20.57 b
*T* (d)	39.35 ± 0.47 a	38.76 ± 0.53 a	36.42 ± 0.65 b

## Data Availability

The data presented in this study are available on request from the corresponding author.

## Appendix A

Text A1 Bacterial 16S rDNA V3-V4 hypervariable region amplification conditions

The bacterial 16S rDNA V3-V4 hypervariable region (ABI GeneAmp ^®^ 9700 PCR instrument) was amplified by primers 338F (5′-ACTCCTACGGGGAGGCAGCAG-3′) and 806R (5′-GGACTACHVGGGTWTCTAAT-3′) with barcode. The reaction procedure was pre-denaturation at 95 °C for 3 min, denaturation at 95 °C for 30 s, annealing at 53 °C for 30 s, extension at 72 °C for 45 s, 30 cycles, and extension at 72 °C for 10 min.

Text A2 Composition of culture medium substances

MRS: casein peptone 10 g, beef extract 10 g, yeast extract 5 g, glucose 5 g, sodium acetate 5 g, diamine citrate 2 g, Tween 80 1 g, K_2_HPO_4_ 2 g, MgSO_4_·7H_2_O 0.2 g, MnSO_4_·H_2_O 0.05 g, CaCO_3_ 20 g, and agar 15 g. The pH was adjusted to 7.0 with 5 mol/L NaOH, and the volume was fixed to 1 L.

NA: peptone 10 g, beef extract 10 g, NaCl 5 g, and agar 15 g; adjust pH to 7.0 with 5 mol/L NaOH, with a constant volume of 1 L.

The above liquid medium was prepared without agar and autoclaved at 121 °C for 20 min.

Text A3 Bacterial liquid treatment

The single colony of the obtained strain was deposited into a 50 mL centrifuge tube (containing 30 mL liquid medium), shaken at 30 °C for 10 h, centrifuged at 500 rpm for 3 min, and the supernatant was discarded. After adding 30 mL PBS buffer (PH = 7.4, 137 mM NaCl, 2.7 mM KCl, 10 mM Na_2_HPO_4_, 2 mM KH_2_PO_4_, 10 mM), it was blown evenly, and 3 μL was taken on the Tangmai blood cell counting plate (Beijing Solaibao Technology Co., Ltd., Beijing, China) to calculate the number of bacteria, repeated 3 times, and the concentration was diluted with PBS buffer to 1 × 10^8^ cfu/mL.

**Table A1:** Summary of Illumina HiSeq sequence statistics for samples.

Sample\Info	Seq_Num	Base_Num	Mean_Length
O_1	53,374	22,754,724	426.326001
O_2	42,753	17,989,920	420.787313
O_3	46,078	19,347,247	419.880355
P_1	47,901	20,298,042	423.749859
P_2	41,871	17,727,270	423.378233
P_3	52,861	223,63,693	423.066022
A_1	35,478	15,061,449	424.529258
A_2	46,772	19,991,738	427.429616
A_3	46,207	19,455,154	421.043435
C_1	37,383	15,880,802	424.813471
C_2	40,793	17,313,286	424.418062
C_3	39,855	16,885,452	423.672111

Note: The first column is sample name, the second column is number of optimized sequences, the third column is number of optimized bases, and the fourth column is average length of sequences. Different capital letters represent different diets: O, orange-feeding; P, peach-feeding; A, apple-feeding; C, cucumber-feeding.
